# Cryoanalgesia and Lung Isolation: A New Challenge for the Nuss Procedure Made Easier With the EZ-Blocker™

**DOI:** 10.3389/fped.2021.791607

**Published:** 2021-11-29

**Authors:** Nicole McCoy, Laura Hollinger

**Affiliations:** ^1^Anesthesia and Perioperative Medicine, Medical University of South Carolina, Charleston, SC, United States; ^2^Department of Surgery, Division of Pediatric Surgery, Medical University of South Carolina, Charleston, SC, United States

**Keywords:** Nuss, cryoanalgesia/cryoablation, pediatric, bronchial blockade, pectus excavatum

## Introduction

Pain control modalities for minimally invasive repair of pectus excavatum (Nuss procedure) are evolving. While thoracic epidurals, regional anesthesia catheters, and patient-controlled analgesia effectively treat post-operative pain, each of these modalities bears their own unique set of benefits and challenges. In lieu of these, intercostal nerve cryoanalgesia has recently been adopted by pediatric surgeons performing the Nuss procedure, demonstrating decreased length of stay, hospital cost, and narcotic consumption as well as improved long-term pain control (1). Specifically, cryoanalgesia has been shown to provide equivalent analgesia to thoracic epidurals without the risks of epidural placement (2).

## Background

Cryoanalgesia is performed thoracoscopically by holding direct contact of the cryoprobe onto each intercostal nerve for a designated time period during extreme cooling, with additional time for defrosting and safe probe removal. The second through seventh intercostal nerves are typically targeted, or those which best correlate to the operative area. The cryoprobe is placed lateral to the parasympathetic chain and also excludes the first intercostal nerve to avoid developing Horner's syndrome. The probe cools to −40 to −65°C to ablate the distal nerve while leaving its essential framework intact for future regeneration. Lung isolation and controlled deflation are necessary to provide adequate exposure as well as prevent inadvertent lung injury by the actively cooled cryoprobe, adding a unique twist to a previously straightforward airway technique. We find bilateral trocar placement to be essential to deliver both the thoracoscope and the cryoprobe, as its extreme cold temperature needs to be protected from the patient's skin. Similar to others, we deliver these trocars through our standard Nuss procedure incisions, with the addition of a 5 mm trocar incision as necessary depending on patient anatomy (2). The Nuss procedure is performed in standard fashion.

Airway management options include placement of a double lumen endotracheal tube or bronchial blocker to achieve appropriate exposure. A double lumen endotracheal tube can safely and quickly achieve lung isolation but may be challenging in younger, smaller patients. Risks of double lumen tube placement include sore throat, hoarseness, and airway trauma (3, 4). Our institution has adopted the use of the EZ-Blocker™Endobronchial Blocker (Teleflex, Inc., Wayne, PA, USA) for lung isolation in an effort to reduce these untoward effects while providing adequate surgical exposure.

## Discussion

Cryoanalgesia is typically preformed thoracoscopically and, in our practice, prior to correction of the pectus defect. Lung isolation is facilitated by the EZ-Blocker™and reinforced with intrathoracic insufflation pressure maintained during thoracoscopy. With the lung selectively deflated on the operative side, the probe is held in place for a number of minutes on each nerve while it cools to the designated temperature. It is important to avoid contact with the lung parenchyma while the probe is cooled to prevent inadvertent lung injury, which can result in pneumothorax or bleeding.

The benefits of EZ-Blocker™placement for lung isolation include use of a single lumen endotracheal tube, ease of placement due to the bifurcated cuffs, and option for removal after lung isolation is complete. In our group, despite minimal provider experience with placement of this novel blocker, we encountered no issues with blocker dislodgement or inadequate surgical exposure. In 2018, a retrospective review concluded successful and stable lung isolation in a majority of patients 6 years of age and older when the EZ-Blocker™was placed extraluminally (5). Our patients were 12 years of age and older; therefore, a standard intraluminal approach through a 7.0 endotracheal tube was used for placement.

To maximize success, here are a few lessons learned from EZ-Blocker™use in our patient cohort. Attempt to keep your endotracheal tube midline during blocker placement. This allows for improved deployment of the bifurcated cuffs as they exit the endotracheal tube. Avoid water-based lubricants; opt instead for silicone-based spray to prevent the cuffs from drying and sticking to one another upon deployment. A fiberoptic scope should be used to confirm placement of the blocker straddling the carina and adequate cuff inflation. Lung deflation may take longer than expected, as compared to a double lumen endotracheal tube, due to the small channel within the blocker—have patience and ensure you have removed the cap for proper egress of air.

The use of the EZ-Blocker™should be considered as a safe, effective, and efficient alternative to double lumen endotracheal tube placement in children undergoing minimally invasive repair of pectus excavatum. We encourage you to have confidence with this novel variation of the bronchial blocker as your surgeon requests lung isolation for intercostal nerve cryoanalgesia.

## Author Contributions

All authors listed have made a substantial, direct, and intellectual contribution to the work and approved it for publication.

## Conflict of Interest

The authors declare that the research was conducted in the absence of any commercial or financial relationships that could be construed as a potential conflict of interest.

## Publisher's Note

All claims expressed in this article are solely those of the authors and do not necessarily represent those of their affiliated organizations, or those of the publisher, the editors and the reviewers. Any product that may be evaluated in this article, or claim that may be made by its manufacturer, is not guaranteed or endorsed by the publisher.
